# Supplementary material for: Obesity in young South African women living with HIV: A cross-sectional analysis of risk factors for cardiovascular disease
Source: PLoS One. 2021 Nov 15;16(11):e0255652. doi: 10.1371/journal.pone.0255652 (PMC8592426; doi:10.1371/journal.pone.0255652)

Integration of Cardiovascular Disease Screening and Prevention in the HIV Management Plan for Women of Reproductive Age in a Resource-limited setting

**The ISCHeMiA study**

**Protocol version 1.0 dated 1 Aug 2018**

**STUDENT NAME:** Sherika Hanley

**STUDENT NUMBER:** 993225740

**COURSE NAME:** Doctor of Philosophy (Medicine)

**DISCIPLINE:** Internal Medicine

**COURSE CODE:** MEDI9NT1

**SUPERVISOR:** Dhayendre Moodley

**CO-SUPERVISOR:** Mergan Naidoo

**CONTENTS**

BACKGROUND 4

Research Question/Hypothesis 5

AIM and objectives 6

Overall Aim 6

Specific Objectives 6

LITERATURE REVIEW 7

Introduction: 7

HIV and Dyslipidamia 7

HIV and Hypertension 8

HIV, obesity and insulin resistance 8

Other risk factors for CVD 9

CVD risk assessment tools in HIV 9

CVD risk assessment in young adults 10

Measurements of subclinical atherosclerosis 10

Current CVD risk modification in HIV 10

Rationale 11

conceptual framework 12

research design and methods 14

Overview 14

Ethical Considerations 21

Work Plan 22

Budget 22

Study period / Time lines: 23

References 24

Appendices Data Collection Tool – Intervention 27

Data Collection Tool – Control 28

# BACKGROUND

Worldwide, there are 36.7 million people living with HIV (PLWH) with a marked increase in uptake of antiretroviral therapy (ART) and a steep decline in numbers of AIDS-related deaths. However globally the leading cause of death is from cardiovascular disease (CVD) with the majority of deaths occurring in developing countries. The path to atherosclerotic CVD progression, mainly coronary artery and cerebrovascular disease, remains silent until advanced stages and the end result is significant disability or death.

An estimated 16.6% of South Africans aged between 15-49 years are living with HIV, of which 23.2% are women of reproductive age. A reduction in HIV-related mortality among women of reproductive age in South Africa is largely attributed to increased access to antiretroviral treatment resulting in non-communicable diseases such as diabetes, cerebrovascular disease and hypertension surpassing HIV as the leading causes of death. Although there appears to be higher risk of CVD in HIV infected men, international studies have shown that the risk of CVD in HIV infected women is significant. As more HIV infected South African women of reproductive age access antiretroviral treatment resulting in potential increased life expectancy, there remains a paucity of data for non-communicable co-morbidities in this vulnerable population group.

In addition to the well-recognized traditional risk factors for CVD in HIV uninfected women, there is increasing evidence of HIV-related chronic inflammation and immune activation and certain antiretroviral drugs that contribute to the increased presence of metabolic syndrome in PLWH. The presence of metabolic syndrome defined by elevated blood pressure, glucose, dyslipidaemia and abdominal obesity, doubles the risk of cardiovascular disease. A systematic review has shown that the mean prevalence of metabolic syndrome among PLWH in Africa was 30.5%. Local studies have demonstrated an increase in obesity in the first 3 years after HIV acquisition and that obesity is more pronounced in black females compared to males. Investigators have further demonstrated that South Africa is facing an epidemic of hypertension, and that there is a high prevalence of concurrent hypertension and HIV highlighting the need for improved integration of management guidelines. The Heart of Soweto study has shed light on the rising incidence of acute coronary syndrome and other cardiovascular diseases in Black South Africans.

Several studies have shown that majority of the non-nucleoside reverse transcriptase inhibitors (NNRTIs), including current 1^st^ line ART Efavirenz, and NRTIs, currently in use are associated with impaired lipid metabolism whereas the newer ART have not been associated with significant adipocyte dysfunction.

Methods of risk prediction and risk reduction strategies for atherosclerotic CVD in people living with HIV remain a major research gap in developing countries. A recent recommendation by the World Health Organization (WHO) is the provision of a cardiovascular risk assessment in all people living with HIV using standard protocols recommended for the general population. The commonly used cardiovascular risk assessment tools are non-specific to HIV and/or resource-constrained settings. The South African dyslipidaemia guidelines advocate CVD risk assessment at ART initiation by means of the Framingham table which underestimates risk in this population and is non-specific to LMIC. WHO Package of Essential Non-communicable Disease interventions for primary health care in low resource settings (WHO PEN) encompasses the WHO and International Society of Hypertension (WHO/ISH) cardiovascular risk prediction charts which are specific to 14 epidemiological sub-regions, and applying charts for sub-region Afr E would be a more suitable CVD risk assessment tool in our setting. In addition to the risk assessment, the WHO PEN provides concise management guidelines for CVD risk modification.

The association between HIV and CVD risk factors in South Africa has been documented mainly in cross-sectional studies. The WHO PEN is the investigator’s choice of intervention in this proposed prospective study in women who receive HIV care. There are currently no studies assessing the impact of WHO PEN interventions in people living with HIV in resource-constrained settings.

## Research Question/Hypothesis

An integrated model of HIV care, that includes the WHO PEN, will identify and modify risk factors in more women when compared to a HIV management plan that does not include screening and prevention of atherosclerotic cardiovascular disease.

# AIM and objectives

## Overall Aim

The overarching aim of the study is to determine the impact of including screening for prognostic markers of cardiovascular disease on the modification of cardiovascular risk factors in HIV positive women on ART.

## Specific Objectives

**Primary objectives:**

- Determine the effectiveness of the *WHO Package of Essential Non-communicable Disease interventions for primary health care in low resource settings* (WHO PEN), integrated with standard of care HIV management guidelines, in identifying and in modifying risk factors for atherosclerotic cardiovascular disease in women receiving HIV care.
- Compare cardiovascular disease (CVD) risk scores using the WHO PEN *WHO/ISH Cardiovascular risk prediction chart for WHO epidemiological sub region AFR E*, *Data collection on adverse effects of anti-HIV drugs study (DAD risk assessment) and Framingham risk assessment* with short-term cardiovascular disease outcomes (presence of subclinical atherosclerosis by means of CMIT, presence of CVA, Myocardial infarction, Angina) in women aged over 40 years.

**Secondary objectives:**

- Compare CVD risk between women receiving Efavirenz versus Lopinavir/r containing ART regimens.

# LITERATURE REVIEW

## Introduction:

A Sciencedirect and Pubmed literature search of multi-national clinical trials, observational studies and systematic reviews all allude to the increasing incidence of cardiovascular disease (CVD) in HIV uninfected and infected populations in developed and developing nations, and in young and old([2-7](#_ENREF_2)). Traditional risk factors for CVD, namely genetic predisposition and behavioural risk factors such as the use of tobacco, excessive alcohol consumption, sedentary lifestyle, and inappropriate diet are well known in the general population, and are becoming more evident in people living with HIV (PLWH) in low and middle income countries (LMIC) due to poverty and emerging factors such as increased urbanization([8](#_ENREF_8)).

In African regions and other low and middle-income countries the increased access to antiretroviral treatment and treatment success is unfortunately accompanied by a rise in cardio-metabolic disease ([3](#_ENREF_3), [4](#_ENREF_4), [9](#_ENREF_9)). In addition to known traditional risk factors, HIV through direct mechanisms of persistent immune activation and chronic inflammation in an ageing treated HIV-infected population, and indirectly through the effects of antiretroviral treatment (ART), contribute to the increased presence of intermediate risk factors of CVD in PLWH ([5](#_ENREF_5), [10](#_ENREF_10)). The intermediate risk factors, namely elevated blood pressure and blood glucose, dyslipidaemia and abdominal obesity, make up an entity called metabolic syndrome and the presence of this syndrome doubles the risk of CVD. A systematic review has shown that the mean prevalence of metabolic syndrome among PLWH in developing countries was 30.5%, 21.5%, and 21.4% in Africa, Asia, and South America, respectively. The most common metabolic abnormality across all countries in this review was the presence of a low high-density lipoprotein ([6](#_ENREF_6)).

## HIV and Dyslipidamia

HIV-infected Black Africans, irrespective of ART use, have significantly higher total cholesterol (TC) to High density lipoprotein-cholesterol (HDL), and triglyceride to HDL-C ratios, compared with the HIV uninfected persons ([11](#_ENREF_11)). Untreated PLWH have low HDL cholesterol levels and high triglycerides depicted in a systematic review([12](#_ENREF_12)). South African guidelines on the management of lipid disorders in HIV infected individuals advocate a full lipogram and assessment of cardiovascular risk prior to initiation of ART ([13](#_ENREF_13)) however this is not routinely practiced. According to these guidelines, if elevated lipids are present, the most suitable ART should be selected and watchful use of statins or fibrates may be considered. In the event of dyslipidaemia requiring medical treatment in PLWH on a Protease inhibitor (PI), it is recommended to substitute the PI with an integrase inhibitor or other alternate cholesterol lowering agents to prevent adverse events such as myopathy. However, various studies now demonstrate that not only PIs, but the majority of the Non-nucleoside reverse transcriptase inhibitors (NNRTIs) and NRTIs currently in use are associated with impaired lipid metabolism whereas the newer antiretroviral agents have not been associated with significant adipocyte dysfunction ([5](#_ENREF_5), [14](#_ENREF_14)).

One of the most widely used ARVs in developing countries, Efavirenz (EFV), has been shown to adversely influence cholesterol levels by increasing TC, Low density lipoprotein (LDL) cholesterol, and triglycerides, and impair endothelial cell function, leading to the development of atherosclerosis ([15-18](#_ENREF_15)). Tenofovir disoproxil fumarate (TDF) - based ART had lower lipids compared with other NRTI([14](#_ENREF_14)). Dolutegravir (DTG) use caused no significant change in lipids in the SPRING-1 study ([19](#_ENREF_19)). With regards to management of lipid disorders, the European AIDS Clinical Society (EACS) as do many other guideline committees, advocate initiation of lipid lowering treatment if there is no improvement in cholesterol levels despite a modified diet ([20](#_ENREF_20)). There is an important role for treatment with statins in HIV infection and it is vital to be aware of the indications and contraindications to specific statin therapy within the context of HIV ([21](#_ENREF_21), [22](#_ENREF_22)).

## HIV and Hypertension

Investigators have demonstrated that South Africa is facing an epidemic of systemic hypertension ([23](#_ENREF_23)), another intermediate risk factor of CVD, and what is also been significantly demonstrated is a high prevalence of concurrent hypertension and HIV locally and internationally ([24-27](#_ENREF_24)). It has been shown that PLWH who are not on ART and persons treated with integrase inhibitors had lower systolic BPs than persons who were not on integrase inhibitors([28](#_ENREF_28)). Less than half of patients treated for HIV who had concurrent hypertension received treatment for hypertension in the HOPS study population ([29](#_ENREF_29)) and locally 40% of PLWH were uncontrolled on hypertensive therapy ([30](#_ENREF_30)), demonstrating the suboptimum management of the co-morbidity, hypertension, in PLWH.

## HIV, obesity and insulin resistance

Worldwide and locally, there is evidence of increased obesity in high HIV endemic regions. The presence of obesity defined by a body mass index (BMI) equal to and above 30 kg/m2 increased from 34.4% to 47% in the first 3 years after HIV acquisition in a Durban study ([31](#_ENREF_31)). Locally it has been shown that obesity is markedly increased in South African black females compared to males ([26](#_ENREF_26), [32](#_ENREF_32)). Obesity and adipokine imbalance in PLWH have been shown to contribute to glucose disorders resulting in an incline in the prevalence of diabetes mellitus and other disorders of insulin-glucose homeostasis in PLWH ([33](#_ENREF_33), [34](#_ENREF_34)). The use of ART can lead to insulin resistance directly at a cellular level and indirectly via lipotoxicity ([35](#_ENREF_35)). The widely utilized NNRTI, Efavirenz, compared to an integrase inhibitor, Raltegravir, led to greater fat gain and has been shown to impair adipogenesis and induce pro-inflammatory cytokine production ([36](#_ENREF_36)).

## Other risk factors for CVD

Microalbuminuria is a well-known independent risk factor for cardiovascular disease though the exact mechanism is unclear ([37](#_ENREF_37)). It is most likely elevated due to chronic inflammation and endothelial dysfunction, which is exacerbated by HIV and ART. Measuring high sensitivity C-reactive protein (hsCRP) has been shown to add value to models predicting CVD events in HIV and was demonstrated to be an independent predictor of heart attack and stroke in women ([38](#_ENREF_38)). Levels less than 1, 1 to 3, and greater than 3 mg/L suggest lower, moderate, and high relative risk of CVD. EFV has been associated with a higher increase in hsCRP compared to other ART ([39](#_ENREF_39)).

## CVD risk assessment tools in HIV

A recent recommendation by WHO is the conduct of a cardiovascular risk assessment in PLWH ([40](#_ENREF_40)). The commonly used cardiovascular risk assessment tools do not consider HIV infection, ART type, duration of ART and younger age of PLWH. Cardiovascular risk has therefore been underestimated in PLWH especially younger PLWH ([7](#_ENREF_7)). The most widely used Framingham cardiovascular risk assessment tool has been to some extent, successfully used in PLWH in high income settings, however this tool has does not account for LMIC, and are most often administered in older persons with already well established disease ([41](#_ENREF_41)).

The Data collection on Adverse effects of anti-HIV Drugs (DAD) study includes a coronary heart disease (CHD) equation that calculates the absolute 5-year risk of CHD in PLWH. The DAD equation was developed in high-income countries and utilizes conventional risk factors and ARTs, indinavir, lopinavir/r and abacavir exposure to predict risk of CVD ([42](#_ENREF_42)). In a local study the DAD risk equation demonstrated a 31.1% moderate to high 5 year CVD risk, whereas when applying the Framingham equation only 6.7% moderate to high 10 year CVD risk was seen. Most participants had a low CVD risk by both risk equations in this cross-sectional study in rural SA([30](#_ENREF_30)).

The WHO PEN assesses CVD risk by means of the WHO and International Society of Hypertension (WHO/ISH) cardiovascular risk prediction charts specific to 14 epidemiological sub-regions ([43](#_ENREF_43), [44](#_ENREF_44)). These guidelines have been developed in order to identify people who are at a 10 year risk of developing a major cardiovascular event based on age, gender, systolic blood pressure, presence of diabetes mellitus, total blood cholesterol and tobacco use. Risk assessment begins at age of 40. CVD risk may be higher than indicated by the charts in persons already on antihypertensive therapy, who are obese and who have a sedentary lifestyle. Family history of premature CHD or stroke, elevated triglycerides, low HDL, elevated C-reactive protein, presence of microalbuminuria, tachycardia, fasting glycaemia or impaired glucose tolerance, and socioeconomic deprivation may also indicate a higher risk. Being specific to sub-regions, the WHO/ISH cardiovascular risk is a more suitable CVD risk assessment tool in our setting. Studies assessing the WHO PEN intervention in the general population have shown that it is a feasible management plan in reducing cardiovascular risk([45](#_ENREF_45)). However HIV risk is not factored in and the age of risk assessment should ideally begin at a younger age.

## CVD risk assessment in young adults

The large prospective CARDIA study has shown that young adults with detectable risk factors at baseline, were 2 to 3 times as likely to have coronary artery plaque calcification suggesting that intervention when risk factor levels reach management guideline thresholds may be too late in preventing CVD ([46](#_ENREF_46)). In a large South African study, the prevalence of multi-morbidity (MM) namely HIV, Diabetes and Hypertension in HIV-infected persons in the 18–35 and 36–45 age groups was higher than the patients not on ART, with a higher MM prevalence in the youngest age group in females versus male patients ([24](#_ENREF_24)). HIV and ART cause premature ageing, reiterating the need for an earlier risk assessment and primary intervention ([47](#_ENREF_47)).

## Measurements of subclinical atherosclerosis

There is an increased prevalence of subclinical atherosclerosis in HIV infected people compared to HIV uninfected([48](#_ENREF_48)). Measurement of carotid intima media thickness (CIMT) by ultrasound is a non-invasive method of detecting subclinical atherosclerosis. In a local study of predominantly women, there was a 12% prevalence of subclinical atherosclerosis (CIMT >0.78 mm) which was associated with traditional risk factors of CVD rather than HIV-specific factors ([49](#_ENREF_49)).

## Current CVD risk modification in HIV

Despite all that is known, there is a lack of integration in current HIV management and cardiovascular prevention/management guidelines in South Africa. Integration of HIV and Non-communicable diseases (NCD) testing in rural Uganda and Lesotho identified a high number of undiagnosed HIV, hypertension and diabetes([50](#_ENREF_50)). Chronic care models in HIV supported by USAID have been implemented in Kenya, Nigeria and Zambia ([41](#_ENREF_41), [51](#_ENREF_51)). In South Africa, a good way to start from a practicality view point is the Essential Drug Lists (EDL PHC). The EDL has dedicated chapters on both management categories all in one convenient reference guide. However the patient receiving treatment at the HIV clinic usually has measurements of basic vital signs, for example, height, weight, temperature and blood pressure. If an abnormality is detected, they are usually referred to a different department or institution for the management of non-communicable diseases, in a silo fashion. Current local HIV guidelines recommend a blood pressure (BP) measurement, urinalysis for glycosuria, serum creatinine at HIV diagnosis and ART initiation. A fasting cholesterol and triglycerides blood is analysed if the individual requires Lopinavir or other PIs. Thereafter there is no recommendation for screening of BP, glucose, cholesterol and other risk factors of cardiovascular disease (CVD) in the follow-up plan of persons on antiretroviral therapy (ART) in the National HIV treatment guidelines. There is minimal data available assessing the outcomes in PLWH who receive a comprehensive integrated HIV and NCD treatment package and there are no studies comparing institutions who provide integrated treatment versus compartmentalized treatment. The WHO ICCC model and SA Integrated Chronic Disease Management are proposed frameworks currently not implemented ([52](#_ENREF_52)).

## Rationale

South Africa has begun rolling out the Universal test and treat guidelines attempting to valiantly halt the high incidence of HIV. However despite longer life expectancy, PLWH have a higher risk of death compared to HIV uninfected persons due to presence of NCDs, especially from atherosclerotic CVD. The main emphasis of management in South Africa and other low and middle-income countries is on communicable diseases and there is a lack of reinforcement of structured chronic non-communicable diseases (NCD) guidelines and monitoring, with scanty statistics on morbidity related to NCDs such as CVD. Cardiovascular disease is the leading cause of death in women and the risk is often underestimated in women([53](#_ENREF_53)). This study proposes to provide data on the prevalence and incidence of CVD risk factors in a cohort of HIV-infected women from a low income background, and highlight potential benefits of a cost-effective, fundamental set of evidence-based interventions for improving morbidity and mortality from CVD and it’s risk factors, that is achievable in LMICs from Primary Health Care (PHC) levels.

# conceptual framework

The conceptual framework for this study is based on the complex web of causes that lead to cardiovascular disease and its endpoints. The study aims to identify interactions between the well-known conventional behavioural, environmental and metabolic causes of CVD with HIV and ART.

Furthermore, the framework for integrated management of chronic multi-morbidity of non-communicable diseases (NCD) and infectious chronic disease (ICD) is based on WHO Innovative care for chronic conditions model (ICCC). The ICCC framework focuses on improving primary healthcare at three levels: micro level (individual and family), meso level (healthcare organisation and community), and macro level (policy). An adaptation of this model is by Oni et al, which incorporates the colliding epidemics of NCDs and ICDs.

MESO

MICRO

MACRO

WHO ICCC Framework modified by Oni et al([1](#_ENREF_1)) and further modified by the investigator for the purposes of this protocol

Policy Environment

Patients and Families

Health Care Team

Community

Partners

# research design and methods

## Overview

1. Study Setting: Umlazi/Philasande Research Clinic and Gateway Umlazi Clinic
2. Study Design: This study is a prospective two-arm, quasi-experimental design comparing a primary health care intervention plan with usual care.
3. Target Population: HIV infected women attending PHC
4. Study population: HIV infected women aged from 18 to 49 years of age and receiving HIV care at PHC clinics.
5. Inclusion criteria

- HIV infected women
- On ART for at least 1 year
- Equal to/older than 18 years of age
- Equal to/younger than 49 years of age
- Plans to remain in the study catchment area for at least 3 years

1. Exclusion criteria

- Participant declines study participation

1. Sampling

• Size of sample

400 HIV-infected women consisting of an intervention arm of 200 women co-enrolled in the PROMOTE study at the Umlazi CRS (see method of sampling for details), and a control arm of 200 women receiving HIV care at the PHC. With a 20-30% estimated prevalence of metabolic syndrome in people living with HIV without any specific intervention in the risk factor modification of cardiovascular disease and a proposed 10-15% estimated incidence of metabolic syndrome in the intervention arm, 132 participants per arm would allow for 80% power. An additional 10% can be added to allow for loss to follow-up. The larger proposed sample size will allow for external validity / generalizability.

- Method of selecting sample

Two HIV-infected female cohorts will be matched for receipt of ART duration >1 year and for age will be compared in the study. Method of selection will be via convenience sampling.

**Intervention group**: There are 245 HIV infected women between 18 and 49 years of age, who have been on ART for more than 1 year and attending the CAPRISA Research Clinic in Umlazi. These women were enrolled into the PEPFAR PROMise Ongoing Treatment Evaluation (PROMOTE) observational study from May-June 2017. This study has been implemented to provide long-term follow-up data on safety outcomes of widespread use of combination antiretrovirals (cART) among an already well-characterized cohort of HIV infected mothers and their children who previously enrolled in the multi-site PROMISE study. This cohort was selected for the proposed intervention arm because the principal investigator is based at the research clinic. Women will be briefed at their next PROMOTE study visit and the 1^st^ 200 interested candidates meeting all eligibility criteria will be co-enrolled into the Intervention arm of the Sub-study.

**Control group**: The Tier data base will be used to select HIV infected women aged between 18-49 years, receiving ART for more than 1 year at the Umlazi Gateway PHC. Scheduled clinic visits at similar time points to the anticipated clinic visits in the intervention group will be used to establish a list of potentially eligible women. Following a matched pool of data, the first 200 women fulfilling the inclusion criteria, who attend the clinic for their next appointment and who consent to study participation will be enrolled.

1. Data sources

- Intervention group: Each participant in the intervention group will be assigned a new Participant identifier number (PID) in addition to the PROMOTE PID. History taking and physical exam, as well as laboratory results and where applicable ultrasound reports, will be entered directly into a data collection tool on a Microsoft Excel spreadsheet (Appendix I).
- Control group: Participants will be assigned a PID in addition to their clinic chart number. Information from clinic patient medical records will be entered directly into the data collection tool (Appendix II). At the final study visit; history, physical exam, lab report finding and ultrasound findings (where applicable), will be source documented.

1. Measures to ensure validity

- Internal
- The prospective nature of the study, large sample size, careful matching between cohorts by age category and by duration of ART aims to counteract the potential for selection bias.
- Information bias in chart review will be controlled by training of staff involved in data collection. Missing information
- Loss to follow up – a retention plan will be place to send clinic appointment reminders by sms or whatsapp, telephone calls in the case of missed visits and occasionally home visits when deemed necessary.
- Laboratory sample measurements will be performed by a certified laboratory.
- External -The larger proposed sample size will allow for external validity / generalizability.

1. List of Variables to be measured and schedule of evaluations

| CVD risk factors | Intervention Arm  (at main study PROMOTE scheduled visits)  Year 1(0+6m), Year 2(12+18m), Year 3(24+30m), Final study Visit (36m) | Control Arm | |
| --- | --- | --- | --- |
|  |  | **Year 1, 2, 3 (not including end of study visit) | Year 3 (end of study-36months) |
| Non-modifiable | Age (Categories in 5 year) | Age (Categories in 5 year) | Age (Categories in 5 year) |
|  | Race (A, I, W, C, O) | Race (A, I, W, C, O) | Race (A, I, W, C, O) |
|  | Family history of CVD in first degree relatives |  | Family history of CVD in first degree relatives |
| Modifiable | Smoking (Current, Never, Past) |  | Smoking (Current, Never, Past) |
|  | Unhealthy Diet |  | Unhealthy Diet |
|  | Exercise (minutes per week) |  | Exercise (minutes per week) |
|  | ART duration in years | ART duration in years | ART duration in years |
|  | ARV regimen | ARV regimen | ARV regimen |
|  | Viral load | Viral load | Viral load |
|  | CD4 count | CD4 count | CD4 count |
|  | BMI (6monthly) | BMI (6monthly) | BMI |
|  | Waist Circumference (6 monthly) |  | Waist Circumference |
|  | Systolic BP (6monthly) | Systolic BP | Systolic BP |
|  | Pulse (6monthly) |  | Pulse |
|  | Fasting Glucose (annual) |  | Fasting Glucose |
|  | Fasting Lipogram (annual) | Fasting Lipogram | Fasting Lipogram |
|  | hsCRP (annual) |  | hsCRP |
|  | Urine Microalbumin (annual) |  | Urine Microalbumin |
|  | *Carotid intima media thickness |  | Carotid intima media thickness |
|  | CVD risk % by Framingham |  | CVD risk % by Framingham |
|  | CVD risk % by DAD |  | CVD risk % by DAD |
|  | CVD risk % by WHO/ISH |  | CVD risk % by WHO/ISH |

*Only at year 3 in women aged over 40 years **Data collection through chart review

1. Plan for Data collection

- **Intervention group**: The prevalence of CVD risk factors will be determined by data collection through history taking, physical examination, and laboratory and radiology investigations as per the data collection tool. CVD risk assessment will be performed annually using a combination of the WHO and International Society of Hypertension cardiovascular risk prediction (WHO/ISH) and the DAD CHD equation. The intervention proposed is a modified WHO PEN algorithm incorporated into HIV management guidelines at study entry. Trends in all risk factors will be monitored, and new risk factors identified, with 6 monthly intervention during the first year, and annually for three years thereafter.
- **Control group**: Following informed consent, data will be collected from the participants ARV clinic medical chart as per the data collection tool. Standard of care HIV management and primary health care will be provided by public sector clinic staff according to current national guidelines. No study investigations or study questionnaires will be carried out at entry visit until the final study visit. There will be regular telephonic follow up with the participants to maintain study retention. Chart review data collection at 6 monthly intervals. CVD risk factors, as per outcomes described above, will be measured by the study at a single 3 year final study visit. Information obtained will be conveyed to the clinic staff for further management if required.

1. Plan for Data handling/processing

- Data will be analysed using SAS or SPSS software

1. Statistical methods

- Descriptive statistics-continuous variables will be represented by means, medians, prevalence, standard deviation.

The categorical variables will be represented by N+%

- Analytic statistics

The continuous variables will be compared by use of t-tests or Wilcoxon rank sum tests.

The categorical variables will be compared using Chi sq or Fisher’s exact test/

- Logistic regression will be applied to identify predictors of cardiovascular disease between the two arms, and between those participants who exhibit atherosclerosis by carotid intima thickness and those who don’t.

1. List of possible confounders

- Women in the intervention group are already enrolled in a study with controlled settings. These women may have commenced ART with a higher baseline CD4 counts.
- Multivariate logression analyses will be utilized in order to control for confounders.

1. List of associations to be measured

|  | BMI | WC | Fasting lipogram | Systolic BP | Fasting  Glucose | Urine microalbumin | hsCRP | Carotid initima medial thickness |
| --- | --- | --- | --- | --- | --- | --- | --- | --- |
| Age | x | x | x | x | x | X | x | X |
| Family History  of CVD | x | x | x | x | x | X | x | X |
| Tobacco use | x | x | x | x | x | X | x | X |
| Diet | x | x | x | x | x | X | x | X |
| Exercise | x | x | x | x | x | X | x | X |
| ART regimen | x | x | x | x | x | X | x | X |
| ART duration | x | x | x | x | x | X | x | X |
| VL | x | x | x | x | x | X | x | X |
| CD4 | x | x | x | x | x | X | x | X |
| BMI |  |  | x | x | X | X | x | X |
| WC |  |  | x | x | X | X | x | X |
| Fasting Lipogram |  |  |  | x | X | X | x | X |
| Systolic BP |  |  |  |  | X | X | x | X |
| Fasting Glucose |  |  |  |  |  | X | x | X |
| U-microalbumin |  |  |  |  |  |  | x | X |
| hsCRP |  |  |  |  |  |  |  | X |
| CVD risk % by Framingham |  |  |  |  |  |  |  | X |
| CVD risk % by DAD |  |  |  |  |  |  |  | X |
| CVD risk % by WHO/ISH |  |  |  |  |  |  |  | X |

1. Calculate cardiovascular risk as per WHO –ISH, DAD and Framingham, annually in intervention group and in the control group at year 3 and compare short term outcomes (presence of subclinical atherosclerosis by means of CMIT, presence of stroke, MI, angina -ischaemic heart disease)

1. Intervention:

**DIETARY CHANGES:** All individuals encouraged to reduce daily salt intake by at least one third and, if possible, to <5 g per day, to eat at least 400 g a day of a range of fruits and vegetables as well as whole grains and pulses and to reduce total fat and saturated fat intake

**PHYSICAL ACTIVITY:** All individuals encourage to do at least 30 minutes of moderate physical activity (e.g. brisk walking) a day, through leisure time, daily tasks and work-related physical activity.

**WEIGHT CONTROL:** All individuals who are overweight or obese should be encouraged to lose weight through a combination of a reduced-energy diet and increased physical activity

# Ethical Considerations

- Permissions needed to conduct the study will be obtained from the University of KwaZulu- Natal Biomedical Research Ethics Committee, Prince Mshiyeni Memorial Hospital, KwaZulu-Natal Department of Health and the PROMOTE protocol team.
- Written informed consent form will be obtained prior to any study procedure.
- All study procedures will be conducted in a manner to protect participant privacy and confidentiality.
- There will be no additional reimbursement in the intervention arm of the proposed sub study. Participants enrolled in the PROMOTE study are reimbursed using PROMOTE funds. There will be no reimbursement in the control arm with the exception of the final study visit, during which additional lab assessments will be carried out during their routine PHC visit. .

#

# References

1. Oni T, McGrath N, BeLue R, Roderick P, Colagiuri S, May CR. Chronic diseases and multi-morbidity - a conceptual modification to the WHO ICCC model for countries in health transition. BMC Publ Health. 2014;14.

2. Alencastro PR, Fuchs SC, Wolff FH, Ikeda ML, Brandao AB, Barcellos NT. Independent predictors of metabolic syndrome in HIV-infected patients. AIDS Patient Care STDS. 2011;25(11):627-34.

3. Ali MK, Magee MJ, Dave JA, Ofotokun I, Tungsiripat M, Jones TK, et al. HIV and metabolic, body, and bone disorders: what we know from low- and middle-income countries. Journal of acquired immune deficiency syndromes (1999). 2014;67 Suppl 1:S27-39.

4. Mutimura E, Crowther NJ, Stewart A, Cade WT. The human immunodeficiency virus and the cardiometabolic syndrome in the developing world: an African perspective. J Cardiometab Syndr. 2008;3(2):106-10.

5. Lake JE, Currier JS. Metabolic disease in HIV infection. The Lancet Infectious Diseases. 2013;13(11):964-75.

6. Naidu S, Ponnampalvanar S, Kamaruzzaman SB, Kamarulzaman A. Prevalence of Metabolic Syndrome Among People Living with HIV in Developing Countries: A Systematic Review. AIDS Patient Care STDS. 2017;31(1):1-13.

7. Boccara F, Lang S, Meuleman C, Ederhy S, Mary-Krause M, Costagliola D, et al. HIV and coronary heart disease: time for a better understanding. Journal of the American College of Cardiology. 2013;61(5):511-23.

8. Oni T, Unwin N. Why the communicable/non-communicable disease dichotomy is problematic for public health control strategies: implications of multimorbidity for health systems in an era of health transition. International Health. 2015;7(6):390-9.

9. Deeks SG, Lewin SR, Havlir DV. The end of AIDS: HIV infection as a chronic disease. Lancet. 2013;382.

10. Nasi M, De Biasi S, Gibellini L, Bianchini E, Pecorini S, Bacca V, et al. Ageing and inflammation in patients with HIV infection. Clin Exp Immunol. 2017;187(1):44-52.

11. van Rooyen JM, Fourie CM, Steyn HS, Koekemoer G, Huisman HW, Schutte R, et al. Cardiometabolic markers to identify cardiovascular disease risk in HIV-infected black South Africans. S Afr Med J. 2014;104(3):195-9.

12. Souza SJ, Luzia LA, Santos SS, Rondo PH. Lipid profile of HIV-infected patients in relation to antiretroviral therapy: a review. Revista da Associacao Medica Brasileira (1992). 2013;59(2):186-98.

13. Klug EQ. South African Dyslipidaemia Guideline Consensus Statement2012.

14. Non LR, Escota GV, Powderly WG. HIV and its relationship to insulin resistance and lipid abnormalities. Translational Research. 2017;183(Supplement C):41-56.

15. Maggi P, De Socio GV, Cicalini S, D'Abbraccio M, Dettorre G, Di Biagio A, et al. Use of statins and aspirin to prevent cardiovascular disease among HIV-positive patients. A survey among Italian HIV physicians. New Microbiol. 2017;40(2):139-42.

16. Faltz M, Bergin H, Pilavachi E, Grimwade G, Mabley JG. Effect of the Anti-retroviral Drugs Efavirenz, Tenofovir and Emtricitabine on Endothelial Cell Function: Role of PARP. Cardiovasc Toxicol. 2017;17(4):393-404.

17. Gupta SK, Slaven JE, Kamendulis LM, Liu Z. A randomized, controlled trial of the effect of rilpivirine versus efavirenz on cardiovascular risk in healthy volunteers. J Antimicrob Chemother. 2015;70(10):2889-93.

18. Gleason RL, Jr., Caulk AW, Seifu D, Rosebush JC, Shapiro AM, Schwartz MH, et al. Efavirenz and ritonavir-boosted lopinavir use exhibited elevated markers of atherosclerosis across age groups in people living with HIV in Ethiopia. Journal of biomechanics. 2016;49(13):2584-92.

19. van Lunzen J, Maggiolo F, Arribas JR, Rakhmanova A, Yeni P, Young B, et al. Once daily dolutegravir (S/GSK1349572) in combination therapy in antiretroviral-naive adults with HIV: planned interim 48 week results from SPRING-1, a dose-ranging, randomised, phase 2b trial. The Lancet Infectious diseases. 2012;12(2):111-8.

20. Ryom L, Boesecke C, Gisler V, Manzardo C, Rockstroh JK, Puoti M, et al. Essentials from the 2015 European AIDS Clinical Society (EACS) guidelines for the treatment of adult HIV-positive persons. HIV medicine. 2016;17(2):83-8.

21. Ou HT, Chang KC, Li CY, Yang CY, Ko NY. Intensive statin regimens for reducing risk of cardiovascular diseases among human immunodeficiency virus-infected population: A nation-wide longitudinal cohort study 2000-2011. Int J Cardiol. 2017;230:592-8.

22. Brown WV, Aberg JA, Aspry KE, Longenecker CT, Myerson M. JCL roundtable: Managing lipid disorders in patients with HIV. Journal of clinical lipidology. 2017;11(1):4-11.

23. Mash B, Fairall L, Adejayan O, Ikpefan O, Kumari J, Mathee S. A morbidity survey of South African primary care. PLoS One. 2012;7.

24. Oni T, Youngblood E, Boulle A, McGrath N, Wilkinson RJ, Levitt NS. Patterns of HIV, TB, and non-communicable disease multi-morbidity in peri-urban South Africa- a cross sectional study. BMC Infectious Diseases. 2015;15(1):20.

25. Xu Y, Chen X, Wang K. Global prevalence of hypertension among people living with HIV: a systematic review and meta-analysis. Journal of the American Society of Hypertension. 2017;11(8):530-40.

26. Clark SJ, Gomez-Olive FX, Houle B, Thorogood M, Klipstein-Grobusch K, Angotti N, et al. Cardiometabolic disease risk and HIV status in rural South Africa: establishing a baseline. BMC Public Health. 2015;15:135.

27. Lloyd-Sherlock P, Ebrahim S, Grosskurth H. Is hypertension the new HIV epidemic? International journal of epidemiology. 2014;43.

28. De Socio GV, Ricci E, Maggi P, Parruti G, Celesia BM, Orofino G, et al. Time trend in hypertension prevalence, awareness, treatment, and control in a contemporary cohort of HIV-infected patients: the HIV and Hypertension Study. J Hypertens. 2017;35(2):409-16.

29. Thompson-Paul AM, Lichtenstein KA, Armon C, Palella FJ, Jr., Skarbinski J, Chmiel JS, et al. Cardiovascular Disease Risk Prediction in the HIV Outpatient Study. Clinical infectious diseases : an official publication of the Infectious Diseases Society of America. 2016;63(11):1508-16.

30. Mashinya F, Alberts M, Van Geertruyden JP, Colebunders R. Assessment of cardiovascular risk factors in people with HIV infection treated with ART in rural South Africa: a cross sectional study. AIDS Res Ther. 2015;12:42.

31. Sobieszczyk ME, Werner L, Mlisana K, Naicker N, Feinstein A, Gray CM, et al. Metabolic Syndrome After HIV Acquisition in South African Women. Journal of acquired immune deficiency syndromes (1999). 2016;73(4):438-45.

32. Malaza A, Mossong J, Bärnighausen T, Newell M-L. Hypertension and obesity in adults living in a high HIV prevalence rural area in South Africa. PLoS One. 2012;7.

33. Trends in obesity and diabetes across Africa from 1980 to 2014: an analysis of pooled population-based studies. International journal of epidemiology. 2017;46(5):1421-32.

34. Peer N, Steyn K, Lombard C, Lambert EV, Vythilingum B, Levitt NS. Rising diabetes prevalence among urban-dwelling black South Africans. PLoS One. 2012;7.

35. Feeney ER, Mallon PWG. Insulin resistance in treated HIV infection. Best Practice & Research Clinical Endocrinology & Metabolism. 2011;25(3):443-58.

36. Rockstroh JK, DeJesus E, Lennox JL, Yazdanpanah Y, Saag MS, Wan H, et al. Durable efficacy and safety of raltegravir versus efavirenz when combined with tenofovir/emtricitabine in treatment-naive HIV-1-infected patients: final 5-year results from STARTMRK. Journal of acquired immune deficiency syndromes (1999). 2013;63(1):77-85.

37. Stehouwer CD, Smulders YM. Microalbuminuria and risk for cardiovascular disease: Analysis of potential mechanisms. J Am Soc Nephrol. 2006;17(8):2106-11.

38. Gilotra TS, Geraci SA. C-Reactive Protein as an Independent Cardiovascular Risk Predictor in HIV+ Patients: A Focused Review of Published Studies. J Clin Med Res. 2017;9(11):891-9.

39. Shikuma CM, Ribaudo HJ, Zheng Y, Gulick RM, Meyer WA, Tashima KT, et al. Change in high-sensitivity c-reactive protein levels following initiation of efavirenz-based antiretroviral regimens in HIV-infected individuals. AIDS research and human retroviruses. 2011;27(5):461-8.

40. World Health Organization. Consolidated guidelines on the use of of antiretroviral drugs for treating and preventing of HIV infection: recommendations for a public health approach- 2nd ed. Geneva2016 [

41. Burke EG, Nelson J, Kwong J, Cook PF. Cardiovascular Risk Assessment for Persons Living With HIV. Journal of the Association of Nurses in AIDS Care. 2012;23(2):134-45.

42. Friis-Moller N, Thiebaut R, Reiss P, Weber R, Monforte AD, De Wit S, et al. Predicting the risk of cardiovascular disease in HIV-infected patients: the data collection on adverse effects of anti-HIV drugs study. Eur J Cardiovasc Prev Rehabil. 2010;17(5):491-501.

43. World Health Organization. Prevention of cardiovascular disease : Pocket Guidelines for Assessment and Management of Cardiovascular Risk : (WHO/ISH cardiovascular risk prediction charts for the African Region) Geneva2007 [

44. World Health Organization. Package of essential noncommunicable (PEN) disease interventions for primary health care in low-resource settings Geneva2010 [Available from: [www.who.int/cardiovascular_diseases/](http://www.who.int/cardiovascular_diseases/)publications/pen2010/en,.

45. Hyon CS, Nam KY, Sun HC, Garg R, Shrestha SM, Ok KU, et al. Package of essential noncommunicable disease (PEN) interventions in primary health-care settings in the Democratic People's Republic of Korea: A feasibility study. WHO South-East Asia journal of public health. 2017;6(2):69-73.

46. Loria CM, Liu K, Lewis CE, Hulley SB, Sidney S, Schreiner PJ, et al. Early Adult Risk Factor Levels and Subsequent Coronary Artery Calcification. The CARDIA Study. 2007;49(20):2013-20.

47. Guaraldi G, Orlando G, Zona S, Menozzi M, Carli F, Garlassi E. Premature age-related comorbidities among HIV-infected persons compared with the general population. Clinical infectious diseases : an official publication of the Infectious Diseases Society of America. 2011;53.

48. Vachiat A, McCutcheon K, Tsabedze N, Zachariah D, Manga P. HIV and Ischemic Heart Disease. Journal of the American College of Cardiology. 2017;69(1):73-82.

49. Schoffelen AF, de Groot E, Tempelman HA, Visseren FL, Hoepelman AI, Barth RE. Carotid Intima Media Thickness in Mainly Female HIV-Infected Subjects in Rural South Africa: Association With Cardiovascular but Not HIV-Related Factors. Clinical infectious diseases : an official publication of the Infectious Diseases Society of America. 2015;61(10):1606-14.

50. Chamie G, Kwarisiima D, Clark TD, Kabami J, Jain V, Geng E, et al. Leveraging Rapid Community-Based HIV Testing Campaigns for Non-Communicable Diseases in Rural Uganda. PLoS ONE. 2012;7(8):e43400.

51. FHI 360. Integration of HIV and noncommunicable disease care [Available from: [www.FHI360.org](http://www.FHI360.org).

52. Oni T, McGrath N, BeLue R, Roderick P, Colagiuri S, May CR, et al. Chronic diseases and multi-morbidity - a conceptual modification to the WHO ICCC model for countries in health transition. BMC Public Health. 2014;14:575-.

53. Volpe M, Uglietti A, Castagna A, Mussini C, Marchetti G, Bellagamba R, et al. Cardiovascular disease in women with HIV-1 infection. International Journal of Cardiology. 2017;241(Supplement C):50-6.

## Appendices Data Collection Tool – Intervention


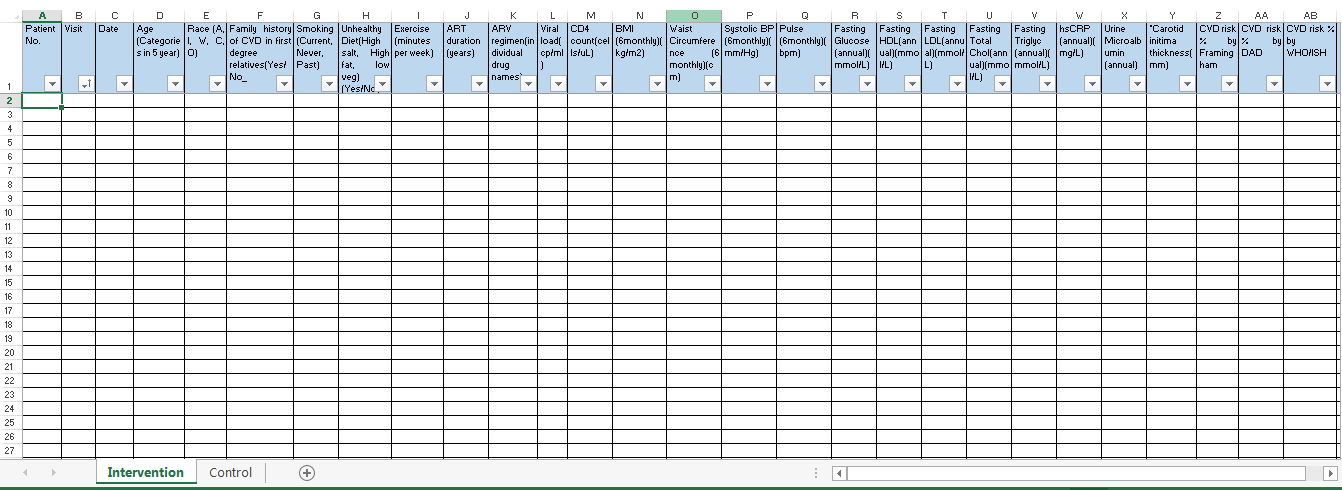


## Data Collection Tool – Control


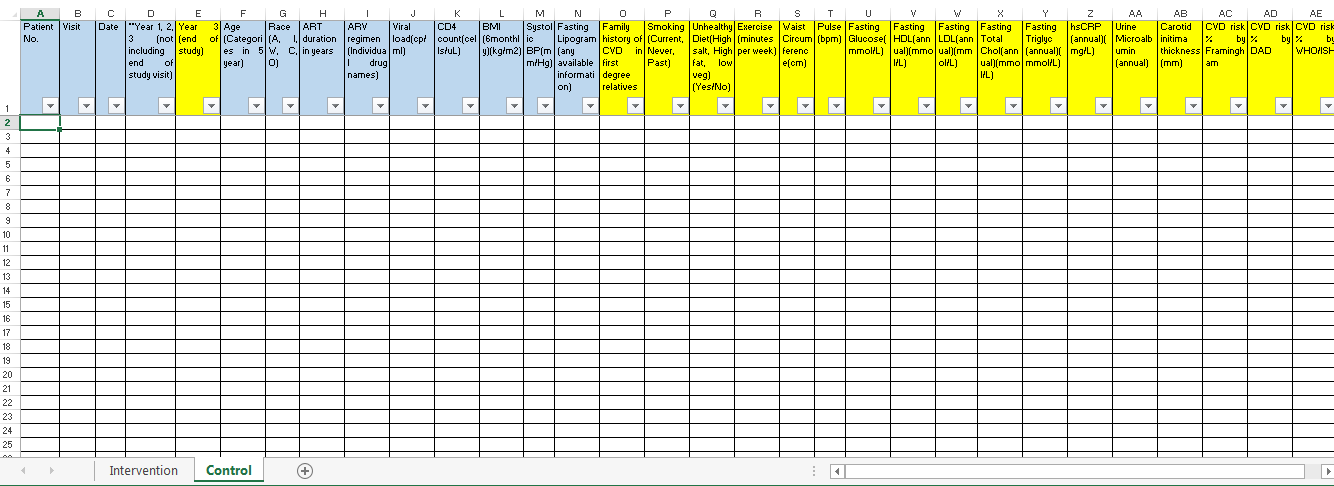

Supplement: S1 File — (DOCX) [file pone.0255652.s001.docx]
