# Supplementary material for: Obesity in young South African women living with HIV: A cross-sectional analysis of risk factors for cardiovascular disease
Source: PLoS One. 2021 Nov 15;16(11):e0255652. doi: 10.1371/journal.pone.0255652 (PMC8592426; doi:10.1371/journal.pone.0255652)
Supplement: S2 File — (PDF) [file pone.0255652.s002.pdf]

ISCHeMiA Protocol Version 1.0 Letter of Amendment #1 dated 26 July 2019

SCHEMA

Title: ISCHeMiA Integration of Cardiovascular Disease Screening and Prevention in the HIV Management Plan for Women of Reproductive Age in a Resource-limited setting

Purpose: Despite longer life expectancy, people living with HIV (PLWH) have a higher risk of death compared to HIV uninfected persons due to presence of Non-communicable diseases (NCDs), particularly atherosclerotic cardiovascular disease (CVD). A recent recommendation by the World Health Organization (WHO) is the provision of a CVD risk assessment in all PLWH using standard protocols. Methods of risk prediction and risk reduction integrated strategies for atherosclerotic CVD in PLHIV, particularly in women who face a dual epidemic of HIV and obesity and who may have culture-specific perceptions of body image, remain a major research gap in developing countries.

Design: A prospective two-arm, quasi-experimental design comparing a primary health care intervention plan with usual care.

Study Population: Women living with HIV aged from 18 to 49 years of age and receiving HIV care at Primary Health Care clinics.

Sample size: About 200 women enrolled in PROMOTE who agree to participate in ISCHeMiA intervention arm, and 200 women from Umlazi Gateway PHC clinic into the control arm.

Study duration: Approximately 3 years

Purpose: This study proposes to provide data on the prevalence and incidence of CVD risk factors in a cohort of women living with HIV from a low and middle income background, and highlight potential benefits of a cost-effective, fundamental set of evidence-based interventions for improving morbidity and mortality from CVD and its risk factors.

Aim:

The overarching aim of the study is to determine the impact of including screening for prognostic markers of cardiovascular disease on the modification of cardiovascular risk factors in HIV positive women on ART.

Primary Objectives

- Determine the effectiveness of the *WHO Package of Essential Non-communicable Disease interventions for primary health care in low resource settings* (WHO PEN), integrated with standard of care HIV management guidelines, in identifying and in modifying risk factors for atherosclerotic cardiovascular disease in women receiving HIV care.

- Compare cardiovascular disease (CVD) risk scores using the WHO PEN *WHO/ISH Cardiovascular risk prediction chart for WHO epidemiological sub region AFR E, Data collection on adverse effects of anti-HIV drugs study (DAD risk assessment) and Framingham risk assessment* with short-term cardiovascular disease outcomes (presence of subclinical atherosclerosis by means of CMIT, presence of CVA, Myocardial infarction, Angina) in women aged over 40 years.

Secondary Objectives

- Compare CVD risk between women receiving Efavirenz versus Lopinavir/r containing ART regimens.
- To assess participant perception of self-body image.
- To evaluate the impact of self-body image perception and other potential barriers to adherence to WHO PEN lifestyle modification interventions

Study Outcomes

- Disease progression to atherosclerosis measured by carotid intima media thickness using ultrasound.
- Body mass index and waist circumference changes over time.
- HsCRP, glucose and lipogram abnormalities based on lab assays.
- Adherence to lifestyle modification advice based on participant report and the above-mentioned study outcomes
